# Supplementary material for: A Novel Method for Rapid and High-Performance SERS Substrate Fabrication by Combination of Cold Plasma and Laser Treatment
Source: Nanomaterials (Basel). 2024 Oct 22;14(21):1689. doi: 10.3390/nano14211689 (PMC11547355; doi:10.3390/nano14211689)
Supplement: Supplementary file 1 [file nanomaterials-14-01689-s001.zip › nanomaterials-3234028-supplementary.pdf]

A Novel Method for Rapid and High-Performance SERS Substrate Fabrication by Combination of Cold Plasma and Laser Treatment

Thi Quynh Xuan Le [1,2], Thanh Binh Pham [1], Van Chuc Nguyen [1], Minh Thu Nguyen [1], Thu Loan Nguyen [1] and Nguyen Thuan Dao [1,2,*]

[1] Institute of Materials Science (IMS), Vietnam Academy of Science and Technology (VAST), 18 Hoang Quoc Viet, Cau Giay, Hanoi 100000, Vietnam
[2] Graduate University of Science and Technology (GUST), Vietnam Academy of Science and Technology (VAST), 18 Hoang Quoc Viet, Cau Giay, Hanoi 100000, Vietnam
* Correspondence: thuandn@ims.vast.ac.vn

Supplementary Information

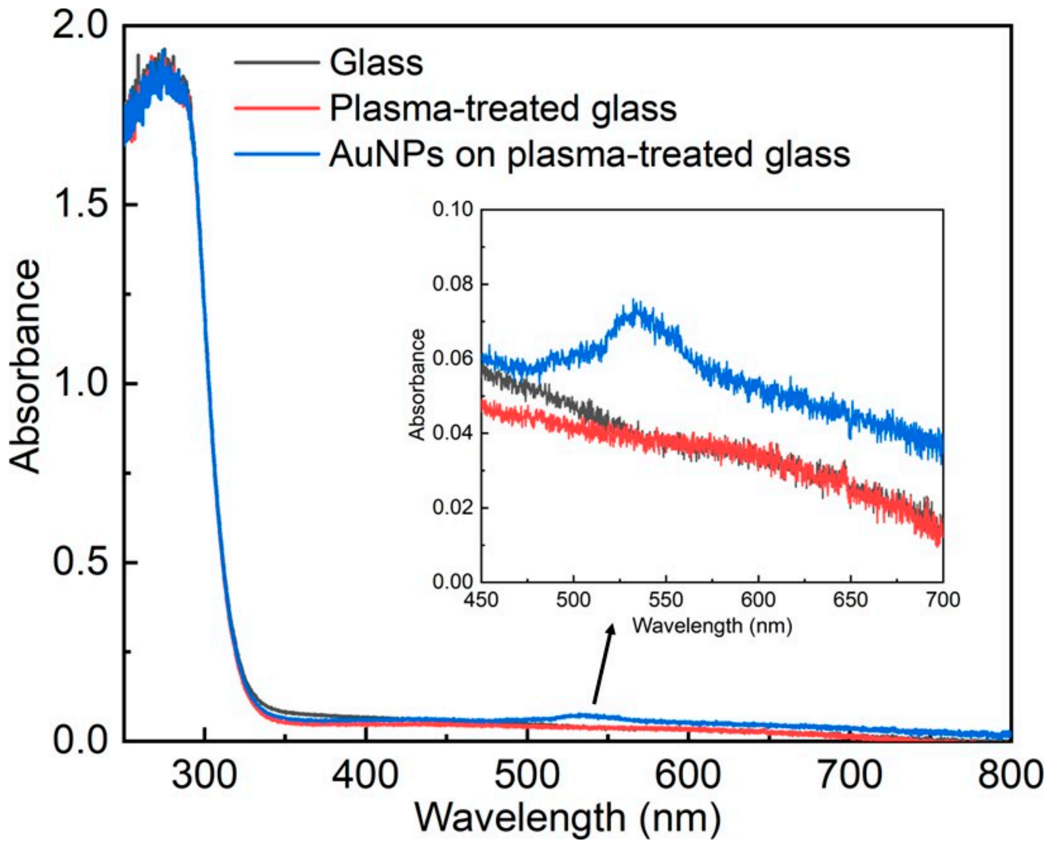

Figure S1. UV-VIS spectra analysis of bare glass, plasma-treated glass, and AuNPs deposited on the plasma-treated glass.

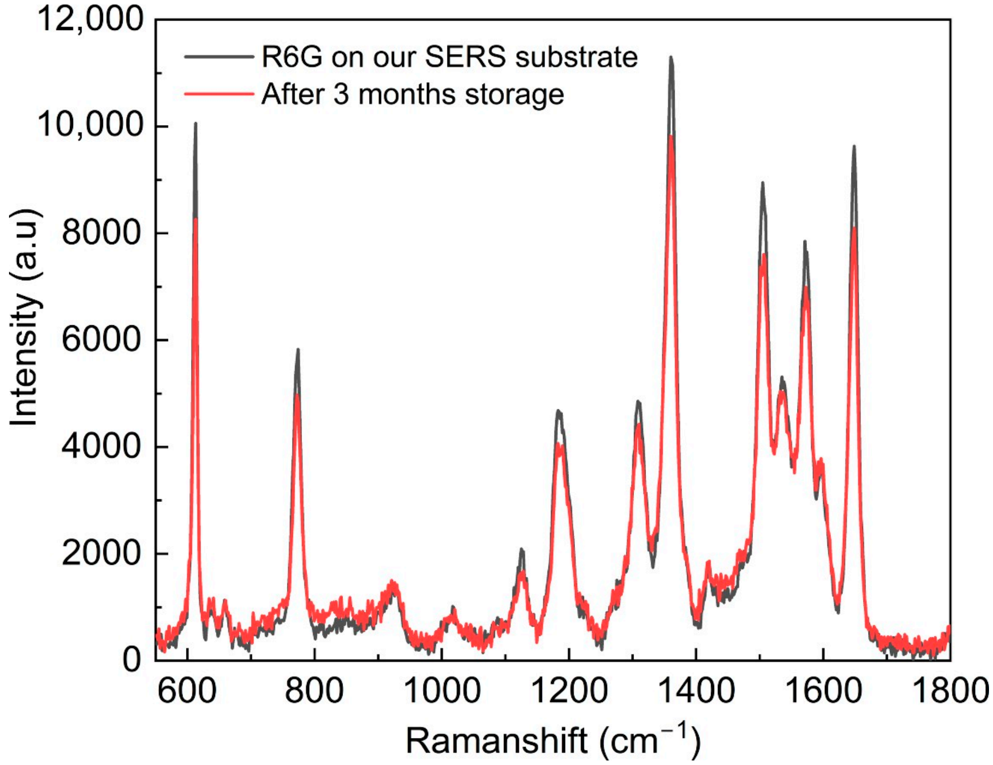

Figure S2. Raman intensity of R6G on our SERS substrate fabricated by combination of cold plasma and laser treatment, before and after 3 months stored at room temperature.
